# Building science and subjectivity from flesh. Towards a reconceptualization of neurophenomenology as a contribution to interdisciplinary health research

**DOI:** 10.1007/s11019-025-10319-x

**Published:** 2026-01-06

**Authors:** Harald A. Wiltsche, Kristin Zeiler

**Affiliations:** 1 Division for Philosophy and Applied Ethics, Linköping University, Linköping, Sweden; 2 Department of Thematic Studies: Technology and Social Change, Centre for Medical Humanities and Bioethics, Linköping University, Linköping, Sweden

**Keywords:** Merleau-Ponty, Flesh, Constitution, Neurophenomenology, Neuroimaging, Qualitative interviews

## Abstract

Radical neurophenomenology offers new ways to re-thinking subjectivity and objectivity, and the relation between them. More specifically, radical neurophenomenology crucially relies on the notion of constitution to circumvent the pitfalls that were associated with older, dualistic frameworks. Building on existing work, our paper has two primary goals. First, we will argue that Merleau-Ponty’s later work provides a suitable framework for elaborating a viable concept of (co-)constitution. As we will discuss, it is especially Merleau-Ponty’s notion of the flesh which allows for a better way to understand the concept of constitution. However, our paper extends beyond purely theoretical analysis and interpretation. Our second goal is to illustrate the pragmatic gain of our clarificatory efforts by discussing how a viable concept of constitution should affect and inform the understanding of modern neuroimaging techniques and a qualitative phenomenological philosophy analysis of subjectivity and lived experience, and how this contributes to neurophenomenological inquiries.

## Introduction

During the last forty years, scholars in phenomenology of medicine have voiced concerns about the dominance of a third-person perspective based on the natural sciences as the primary basis for disease conceptions and diagnostic methods, at the expense of inquiries into lived experiences of illness and pain from the first-person perspective of patients (see, as but some examples, Schwartz and Wiggins 1985; Toombs 1987; Leder 1992; Svenaeus 2019). The rich phenomenological literature on illness, pain, bodily and functional variation, well-being and health can partly be seen as a response to this perceived lacuna (see, again as but some examples, Leder 1990; Carel 2008; Zeiler 2010; Slatman 2016; De Boer et al. 2019; Lajoie 2019). Further, efforts have been made to engage in dialogue across insights from, for example, the neurosciences and phenomenology, for the sake of a better understanding of, i.a., illness, and this in ways that refrain from “naturalizing” phenomenology (see Ratcliffe 2013; Fuchs 2018).

In addition, researchers in the field of neurophenomenology have sought to combine insights from the neurosciences with phenomenology as in, for example, analysis of seizure anticipation, described as possibly contributing to future cognitive therapies (Petitmengin et al. 2007), and analysis of distorted body representation in trauma-related conditions (Rabellino et al. 2016). These last examples illustrate efforts to bring together biomedical third-person inquiries and analysis of research participants’ lived experiences of a particular issue or condition, in what, following Claire Petitmengin (2017), can be referred to as *mild* neurophenomenological studies, within the context of and/or of potential relevance for medicine and health care.

However, the phenomenology of medicine critique has not only centered on developing nuanced accounts of illness. Equally central (and commonly related to the concern with the dominance of the natural sciences) was, and is, the critique of an understanding of subjectivity and objectivity as neatly set apart. This understanding has been seen as part of a mechanistic worldview, and as a Cartesian residue that has profoundly shaped modern medicine (see for example Leder 1992; see also Aho 2019). The critique of this understanding of the relation between subjectivity and objectivity lies at the heart of this article and of *radical* neurophenomenology, to make use of another term by Petitmengin (2017).

Neurophenomenology, as originally described by Francisco Varela, entails a “quest to marry modern cognitive science and a *disciplined approach* to human experience […] in the lineage of the continental tradition of phenomenology” (Varela 1996, p. 330). More specifically, Varela conceived of neurophenomenology as an answer to what Chalmers had labelled the “hard problem” of consciousness, i.e. the problem of explaining how a set of neurons can give rise to subjective experience, and he saw the solution to this problem in a “systematic exploration of the only link between mind and consciousness that seems both obvious and natural: *the structure of human experience itself*” (ibid.) According to Varela, overcoming this problem required moving beyond purely third-person approaches to the mind by incorporating a disciplined analysis of first-person experience. His core idea was that phenomenological and neuroscientific approaches could mutually inform one another—a strategy he called the method of “mutual constraints” (ibid.; Varela 1997; see also Varela et al. 2016). On this view, neuroscientific hypotheses about the correlates of experience should be guided and tested against careful descriptions of lived experience, while those descriptions, in turn, must be refined in light of neuroscientific insights.^1^

Since Varela’s programmatic article originally appeared, neurophenomenology has been developed in various directions. While a detailed overview of the bourgeoning literature is beyond the scope of this article, it is useful to rely on Petitmengin’s distinction between “mild” and “radical” neurophenomenology to distinguish between two broad camps (Petitmengin 2017, p. 142). *Mild* neurophenomenology commonly starts from the uncontroversial fact that “objective information gathered by neuroscientific recordings cannot be interpreted without any reference to subjective experience, to what it feels like to be someone” (ibid.). For instance, to seriously consider whether a particular set of scientific data represents a specific kind of experience, we must have first-personal acquaintance with some of the basic characteristics of that experience. As a result, mild neurophenomenology aims to bridge the gap between subjective experience and physical processes, often by devising procedures to identify “correlations” between experiential descriptions and neural recordings, such as MRIs. In this regard, Varela’s original vision of neurophenomenology can be seen as the blueprint for later manifestations of *mild* neurophenomenology, as when he stated that “only a balanced and disciplined account of both the external and experiential side of an issue can make us move one step closer to bridging the biological mind-experiential mind gap” (Varela 1996: 343).

*Radical* (also sometimes called deep) neurophenomenology, by contrast, is not about identifying correlations between subjective experience and neuroscientific findings nor about bridging the perceived gap. Its aim is instead to “explore the process of *co-constitution* of the objective and subjective poles [that lie] at the root of the hard problem” (Petitmengin 2017, p. 146; our emphasis). This latter approach is also championed by Michel Bitbol who agrees with Petitmengin that instead of trying to correlate the subjective with the objective, (radical) neurophenomenology should be concerned with “an enquiry into the ‘processes of *co-constitution*’ of these domains” (Bitbol 2017, p. 152; our emphasis). As is evident from these two exemplary quotes, the concept of constitution^2^ is pivotal for the project of radical neurophenomenology: it is through the concept of constitution that proponents of radical neurophenomenology aim to *circumvent*, rather than to confront, the hard problem. By ‘circumvention,’ we mean showing that the hard problem dissolves once its underlying dualist presuppositions are exposed as artefactual, rather than attempting to ‘solve’ the problem on its own terms. As put by Bitbol (2012, 165): “[n]o ‘solution’ is given to this [the hard] problem, but a mere ‘dissolution’”. This is done by offering a framework that allows us to recognize the hard problem for what it truly is: a pseudo-problem that evaporates under the right description.

We agree that the hard problem is a pseudo-problem rooted in misguided metaphysical and methodological assumptions and that it can be circumvented with the adoption of an adequate metaphilosophical framework. However, in contrast to Petitmengin and Bitbol, we do not believe that the characteristics of such a framework have been spelled out in sufficient detail yet. We contend that the pivotal concept of constitution requires additional analyses, and that the previous failure to provide these analyses made it challenging to discern the pragmatic gain of radical neurophenomenology. In other words, our concern is not that radical neurophenomenology misidentifies the central challenge, but that it currently still lacks the conceptual resources to fully articulate its proposed remedy.

At the heart of radical neurophenomenology is a deflationary strategy: rather than solving the hard problem, it seeks to *dissolve* it by exposing its ontological presuppositions—chiefly, the dualism between subject and object—as untenable. This reframing relies on a shift in the underlying methodological and metaphysical stance, one that hinges on the concept of co-constitution. However, unless this notion is adequately clarified—especially in ways that avoid revoking mentalistic or subject-centered models of constitution—the project risks undermining its own aim. It is in this context that we turn to the later Merleau-Ponty’s ontology of the flesh, which, we argue, provides the tools to articulate a non-dualist account of constitution suited to the goals of radical neurophenomenology.

In light of these critical claims, we have three aims: First, we argue that Merleau-Ponty’s later work provides a suitable framework for elaborating a viable concept of (co)constitution. Specifically, we suggest that Merleau-Ponty’s notion of the flesh allows for a better way to understand the concept of constitution. Our second aim is to illustrate the pragmatic gain of our clarificatory efforts. We discuss how the radical neurophenomenological framework and the viable concept of constitution should affect and inform *both* the understanding of modern neuroimaging techniques and phenomenological inquiry of research participants’ lived experience, as two domains that are central in much neurophenomenological discussions. In line with our take on radical neurophenomenology, we refrain from the idea of bridging a perceived gap between these domains and instead argue that the reconfigured understanding of constitution offers a basis for a dialogue across neurosciences and phenomenology. Third, while recognizing that much neurophenomenology has focused on the question of the “hard problem” (after all, Varela’s original formulation of neurophenomenology was explicitly designed as a methodological response to Chalmers’ “hard problem”), we note that such work also has researched issues or conditions of relevance for medicine and health care, and brought out its contributions to health care, as in the example of Petitmengin et al.’s (2017) analysis of seizure anticipation. However, while Petitmengin et al.’s 2017 study may be seen as an example of mild neurophenomenology, as it examines “EEG synchronization and subjective correlates” (2016: 758), we argue in this article that the radical neurophenomenology framework via Merleau-Ponty’s concept of constitution *also* matters for research in and of relevance for medicine and health care. Indeed, radical neurophenomenology’s reconfiguration of constitution, along with the rethinking of objectivity and subjectivity renders central concerns that have been crucial in phenomenology of medicine since its inception, namely that of the relation between the natural sciences’ third-person “objective” perspective and the first-person “subjective” perspective. Radical neurophenomenology, precisely through its reconfiguration of constitution and rethinking of objectivity and subjectivity, contributes to these central philosophical questions within medicine and health care.

Thus, it is not only the case that neurophenomenology can contribute to medicine and health care (as mild neurophenomenology does in the case of Petitmengin et al 2017), but radical neurophenomenology, through its re-thinking of central concepts within research in the neurosciences and the phenomenology of medicine—such as that of objectivity and subjectivityvoffer a thorough shift in how to understand these very concepts, and these concepts are central to ongoing debates about the relation between third-person “objective” vs. first-person “subjective” framings in clinical and healthcare contexts.

## From subject to flesh

Our first aim is to demonstrate that it is possible to circumvent the hard problem by adopting a viable concept of constitution, and to assert that Merleau-Ponty’s late philosophy provides the adequate framework for elucidating such a concept. However, there are two fundamental concerns that we must address from the outset.

The first concern is as follows: As mentioned, the hard problem revolves around the question of why certain biophysical mechanisms in our brain are accompanied by subjective experiences or qualia. It’s worth noting that by framing the question this way, one presupposes a dualism that at least initially accepts the ontological distinctness of the realm of consciousness from the realm of biophysical facts. While most mainstream positions directly confront this dualism by either trying to reduce one relatum to the other, or by arguing that the gap between the two relata can be bridged, radical neurophenomenology proposes a deflationary strategy that consists in finding a methodological framework in which the hard problem, together with dualism that underlies it, does not even arise. However, the first concern is that radical neurophenomenology cannot live up to this deflationary goal because of its commitment to the central concept of constitution.

The concept of constitution has a rich history that extends back to at least Immanuel Kant. Kant sought to demonstrate that, crudely speaking, for objects of experience to emerge, raw sense data must be structured through the application of our subjective forms of sensory intuition. However, since the application of these subjective forms of intuition depends on the cognitive faculties, the concept of constitution seems to be linked to subjectivity. While there are, of course, important differences between Kant and Husserl, something analogous appears to be true of how the concept of constitution is understood in phenomenology: Phenomenologically construed, constitution is the name of the process through which an object is given its structure and attributes by the horizontal structure of intentionality.^3^ However, akin to Kant’s philosophy, such a process can appear to be inextricably linked to a robust notion of a conscious subject.

It should now be apparent why one might argue that its commitment to the concept of constitution undermines the aspirations of radical neurophenomenology: On the one hand, radical neurophenomenology seeks to be deflationary by attempting to undercut the subject/object dualism that underlies the hard problem. On the other hand, however, this ambition seems to falter because the concept of constitution—the means by which the direct confrontation with the subject/object dualism is supposed to be prevented—is inextricably linked to one of the relata that constitute the dualism.

The second concern is closely related to the first one. As highlighted in the introduction, our aim is not only to introduce a viable concept of constitution but also to explicate such a concept within the framework of the philosophy of the later Merleau-Ponty, especially with the help of his notion of *flesh*. However, one might be skeptical of this enterprise for reasons very similar to those underlying the first concern. As is well known, Merleau-Ponty developed his philosophy in dialogue with Edmund Husserl’s work. Yet, a certain segment of Merleau-Ponty scholarship portrays “Husserl [as an] exemplar of neo-Cartesian intellectualism” (Dillon 1997, p. 27). Given this reading, it is not surprising that the relationship between Merleau-Ponty and Husserl is sometimes viewed in a predominantly negative light.^4^

Until *Ideas 1*, Husserl’s published work was dominated by a perspective on intentionality as a first-personal directedness towards objects, further distinguishable into intentional acts, meaning contents, and the intended objects. It is this basic structure which was also the canvas for Husserl’s notion of constitution during the *Ideas 1* period: Objects are not simply “out there” but receive their intelligible structure through the sense-bestowing activity of a subject being intentionally directed towards these objects (cf. Husserl 1983; Chap. 2). Following the presentation of phenomenology in *Ideas 1*, Husserl was often portrayed as prioritizing the mental to explicate the physical in terms of it. It is this kind of “intellectualism” that Merleau-Ponty distanced himself from throughout his entire career:^5^ Already in his *Phenomenology of Perception*, *motricity*—a habitualized form of bodily agency—replaces mentalistic interpretations of intentionality as the fundamental layer of human world-involvement. This is the case when Merleau-Ponty emphasizes subjectivity as embodied and embedded in the world (cf. Merleau-Ponty 2002; Young 2005). However, the shift away from accounts explicating intentionality and constitution exclusively by reference to mental activity seems to become even more evident in Merleau-Ponty’s later works: In *The Visible and the Invisible*, Merleau-Ponty (1968) introduces the notion of *flesh* as the conceptual centerpiece of his late philosophy, transforming his philosophy into what seems to be a predominantly ontological program.

It should be apparent why these considerations are relevant in the context of this paper: If it is true that Merleau-Ponty’s late philosophy of the flesh stands in stark contrast with the seemingly mentalistic notion of intentionality, and if it is correct that the notion of constitution requires intentionality as a conceptual canvas, then our aim to spell out constitution within a framework that builds on the later Merleau-Ponty is misguided from the outset.

Our response to these two concerns is as follows: Regarding the first, we agree that for our attempt to circumvent the hard problem to be coherent, we must avoid relying on a conceptual framework that reintroduces the dualism at the heart of the hard problem. This underscores the importance of preventing any contamination of our conceptual apparatus with a mentalistic notion of constitution, as seen in some forms of neo-Kantianism or phenomenology. Yet, in response to the second concern, our stance is more nuanced. As noted, some commentators perceive Merleau-Ponty’s work as a departure from a constitution-based account towards a more thoroughly ontological program. We, however, find this characterization to be inaccurate. We agree with Dimitris Apostolopoulos that instead of breaking completely with the phenomenological tradition before him, Merleau-Ponty’s late philosophy, and especially his “concept of the flesh can plausibly be understood to advance a refined account of intentionality and constitution” (Apostolopoulos 2017, p. 677). It is precisely this characteristic that renders Merleau-Ponty’s late phenomenology appealing for our purposes: it is through Merleau-Ponty’s philosophy of the flesh that we can engage with the notion of constitution without reproducing the dualism that underlies the hard problem.

### Flesh as element

Despite later distancing himself from the approach in the *Phenomenology of Perception* because “I start there from the ‘consciousness’-‘object’ distinction” (Merleau-Ponty 1968, p. 200), a strong anti-mentalistic stance characterizes Merleau-Ponty’s thinking from the start. For instance, when he observes in the *Phenomenology* that “[i]n perception, we do not think the object and we do not think ourselves thinking it” (Merleau-Ponty 2002, p. 277), he rejects a unidirectional act-analysis wherein constitution is viewed as cognitive accomplishment of a conscious, self-transparent mind. Rather than intellectualizing experience in this manner, Merleau-Ponty emphasizes embodiment, affectivity and habitualized coping as the primary means through which we engage our worldly surroundings. “Our own body is in the world”, he writes, “as the heart is in the organism: it keeps the visible spectacle constantly alive, it breathes life into it and sustains it inwardly, and with it forms a system” (Merleau-Ponty 2002, p. 235).

To explicate the flesh, we first turn to the phenomenological distinction between *Leib* or the lived body, i.e., the body as subjectively lived from a first-person perspective, oriented and experiencing in a socio-cultural world, as a relation to and a part of that world, and *Körper* or the body as object. Phenomenologically central, the lived body should not be understood as neatly set apart from the body as an object, but these aspects or dimensions form each other, and lived embodiment comprise both sensing and being sensed, both the body as subjectively lived and as object. Further, even if our embodiment recedes into the background in many everyday activities, as long as we are healthy, it still remains a constant presence: the lived body still remains our pre-reflective center of existence, from and through which we engage with the world. This “inescapability of embodiment” also serves as the entrance point to Merleau-Ponty’s conception of the flesh. Similar to how constitution must not be misunderstood as the animation of raw hyletic data through sense-giving mental acts, it would be equally misguided to conceive of ourselves as embodied subjects standing *before* the world. Engaging with the world is more accurately described as being *in* the world, and, as Merleau-Ponty realized, to be *of* the world, to be of the same basic stuff as the world. The lived body is “a very remarkable variant” of this basic stuff, which Merleau-Ponty calls the original “prototype of Being” (Merleau-Ponty 1968, p. 136). By characterizing our own lived body in this way, as prototypical or carnal Being, Merleau-Ponty underscores the foundational and irreplaceable role of embodiment as a two-fold structure of the lived body/body as object, and as a relation to the world, and of the world. Further, within the same passage from *The Visible and the Invisible*, another pivotal aspect is highlighted—the “constitutive paradox that lies in every visible” (ibid.). To get a better grasp of the nature of this “constitutive paradox”, it is beneficial to explore Merleau-Ponty’s analysis of double-sensations, a phenomenon already elucidated earlier by Husserl.

Given the fundamental role embodiment plays in all our encounters with reality, one might expect the experience of our own body to be one of pure presence and transparency. However, as becomes apparent in the tactile encounter of one of our hands touching the other one, this is far from reality. When our hands touch each other,

there is not just the unidirectional relationship of the one who perceives to what he perceives. The relationship is reversed, the touched hand becomes the touching hand, and I am obliged to say that the sense of touch here is diffused into the body—that the body is a ‘perceiving thing’, a ‘subject-object’. (Merleau-Ponty 1964, p. 166)

This is Merleau-Ponty’s famous *reversibility thesis*. When our hands touch, the experience of our own body is not characterized by pure presence; instead, it is inherently “two-dimensional”, we experience our body as “at once phenomenal body and objective body” (Merleau-Ponty 1968, p. 136). During this experience, the traditional distinctions between subject and object, or between act and object dissolve, revealing the body as “carnal being, as a being of depths, of several leaves or several faces, a being in latency, and a presentation of a certain absence” (ibid.). When we experience our hand being touched and touching, the respective sensations are never completely separate, nor do they collapse into one another. To fully grasp Merleau-Ponty’s position, it is crucial to recognize that the described reversibility is by no means exclusive to the experience of our body. Take as an example the tactile experience of gripping our hands around our car’s steering wheel. We can feel the smooth surface of the leather and exert pressure on the varying textures across different parts of the wheel. But alternatively, we can sense the resistance of the wheel pushing up against our hands, unveiling additional layers of its intricate composition. Like the case of our hands touching each other, our encounter is inherently “two-dimensional”, and our sensations never fully merge yet never entirely disintegrate (Merleau-Ponty 1968, p. 224, 191). What is characteristic, instead, is the reversibility of touching-touched, sensing-sensed, to the extent that Merleau-Ponty views reversibility as *the* defining feature of what he terms as “flesh” (Merleau-Ponty 1964, p. 144). Yet another way to explicate Merleau-Ponty’s understanding of reversibility is to underline, as done by Ted Toadvine, that reversibility in the late Merleau-Ponty does not mean “bi-directionality” (2005: 168). Instead, Toadvine suggests, it can be better understood through the image of “two sides of a coin: existing as a coin requires having both sides, but the two sides are neither equivalent nor interchangeable, and it is impossible for me to see both simultaneously” (2005: 168). Again, this is to move away from dualistic frameworks of subject vs object and instead understand constitution in terms of non-dualistic co-formation that still does not entail a merger and dissolve of differences (and in the latter regard, the image of the coin is limited).

With the above, we are now better positioned to get a sense of Merleau-Ponty’s concept of the flesh. As he emphasizes, the term “flesh” is intended to encapsulate something for which traditional philosophy lacks a name (Merleau-Ponty 1968, p. 139). While explicitly specifying what the flesh is not—neither matter, nor mind, nor substance, nor collective, nor individual—Merleau-Ponty suggests that the flesh can be understood as “the concrete emblem of a general manner of being” (Merleau-Ponty 1968, p. 139). The flesh, as an element, sheds light on “the secret of sensibility”, as Lefort puts it (Merleau-Ponty 1968; lv). It is the fundamental structure present in any segment of being that can ever be experienced or conceived of. As such, it is also the very thing from which every engagement with our environment, every dualism, and every meaningful distinction between relata, springs. It is again apt to return to our description of the body sensing and sensed as not only ambiguously both, but as a “dehiscence, […an] overlapping or encroachment” of the body sentient and sensed (Merleau-Ponty 1968, p. 123). Merleau-Ponty describes the body as “a being of two leaves, from one side a thing among others and otherwise what sees and touches them” (Merleau-Ponty 1968, p. 137), but he immediately corrects himself:

[O]ne should not even say, as we did a moment ago, that the body is made up of two leaves, of which the one, that of the “sensible”, is bound up with the rest of the world. There are not in it two leaves or two layers; fundamentally it is neither thing seen only nor seer only, it is Visibility sometimes wandering and sometimes reassembled. […] To speak of leaves or of layers is still to flatten and juxtapose, under the reflective gaze, what coexists in the living and upright body.

Merleau-Ponty’s central message is clear: To overcome the dualistic frameworks that have constrained our thinking, we must abandon “the age-old assumption that put the body in the world and the seer in the box, or, conversely, the world and the body in the seer as in a box” (Merleau-Ponty 1968, p. 138). Such traditional dichotomies are untenable because it is unclear where to “put the limit between the body and the world, since the world is flesh.” Merleau-Ponty drives his point home by adding that “the world seen is not ‘in’ my body, and my body is not ‘in’ the visible world ultimately: as flesh applied to a flesh, the world neither surrounds it nor is surrounded by it” (Merleau-Ponty 1968, p. 138). Importantly, this implies neither a dualism nor merger or coinciding of body and world or the sentient and the sensible. Making use of metaphors like gap, dehiscence, chiasm and intertwining, Merleau-Ponty seeks to spell out a foundational “identity-and-difference” (Dillon 1997, p. 157) or “identity-in-difference” (Leder 1990, p. 210; Weiss 1999) to capture the relation between subject and object, self and world, sentient and sensible. This makes room for acknowledging “what co-exists” *and* difference, while avoiding traditional dualistic frameworks.

## Constituting (out of) flesh

What renders Merleau-Ponty’s concept of the flesh intriguing for neurophenomenology is the radical reimagining of the relation between subject and object, and consequently, the profound reconfiguration of the concept of constitution. As hopefully is clear from the above, the concepts of reversibility and identity-in-difference are not solely significant when considering the phenomena such as the one of our hands touching and being touched. Reversibility and identity-in-difference also play a crucial role in the experience of non-sentient things. However, in some parts of his oeuvre, Merleau-Ponty extends this notion even further by asserting that a profound form of reversibility permeates *all* encounters with reality, regardless of the sensory modality involved. For instance, in *Eye and Mind*, which compares scientific and artistic experience, Merleau-Ponty notes that “many painters have said *that things look at them*” (Merleau-Ponty 1964, p. 167; our emphasis). He quotes the French painter André Marchand who reports that

[i]n a forest, I have felt many times over that it was not I who looked at the forest. Some days I felt that the trees were looking at me […]. I think that the painter must be penetrated by the universe and not want to penetrate it. I expect to be inwardly submerged, buried. Perhaps I paint to break out. (ibid.)

Merleau-Ponty’s extension of the reversibility thesis to the visual realm was met with skepticism. While Alphonso Lingis concedes a “strange undertone” (Lingis 1986, p. 92) to assertions like “that the seer and the visible reciprocate one another and we no longer know which sees and which is seen” (Merleau-Ponty 1968, p. 139), Dillon reacts more straightforwardly by stating, “I reject this view, [in part] because it is manifestly implausible” (Dillon 1997, p. 161).

It is not our goal here to engage with Lingis’s and Dillon’s analysis in detail. However, it seems safe to say that their discomfort is fueled by the concern that, in the case of visual perception, a fully generalized reversibility thesis might require us “to redefine the verb ‘to see’ in such a way that it becomes meaningful to speak of being, like trees, which see without having eyes [or] to understand human vision in such a way that our eyes are not essential to that seeing” (ibid.). However, in our view, such concerns miss the point, namely that of acknowledging the far-reaching co-constitution of self and world, in a non-dualistic fashion that moves away from attributing activity to the “subject”-side and passivity to the “object” side, and the constitutive interconnectedness of subject and object as thoroughly implicated with each other and emerging through its very interconnectedness. This, we suggest, are points that are central not only in Merleau-Ponty’s discussion of the trees-painter but also, for example, in the summary for the *Passivity* course, as in statements that the “the explication of perceptual experience must make us acquainted with a genus of being with regard to which the subject is not sovereign, without the subject being inserted in it” in a move away from traditional ontologies. Further, this reasoning continues, to “live, for humans, is not merely to impose significations perpetually, but to continue a vortex of experience which is formed, with our birth, at the point of contact between the outside and the one who is called to live it” (Merleau-Ponty 2010, 206). This is not only to bring out how common-sense conceptions of nature are built on the presupposition that objects (such as trees) are “out there”, independently of us, and that we, when we become aware of this, realize that the tree, as it appears to us, already is affected by the peculiarities of our embodied and situated gaze, and by the experiential history that is built into our perceptual horizon. It is also to underline the constitutive, non-dualistic interconnectedness between subject and object. Thus, formulations of trees seeing the painter can be read as an effort to put to words an ontology where not only reversibility is central, but the role of the formative continuous relation between the body and the world for any perception, experience, and knowing, at all.

Where do these considerations leave us regarding the concept of constitution, which, as we have argued, lies at the core of a compelling overall vision of radical neurophenomenology? As Apostolopoulos and others have shown, Merleau-Ponty rejected the idea of constitution as a subjective act of *sense-bestowal*, yet nonetheless remained committed—even in his later work—to developing an account of constitution that addresses what is perhaps the most fundamental phenomenological question: “how phenomena in experience are meaningfully given” (Apostolopoulos 2017, 689). Building on what we have discussed so far about the key concepts shaping Merleau-Ponty’s mature position such as the flesh, we can now outline the main characteristics of a defensible, non-intellectualist notion of constitution.

As noted earlier, any attempt to circumvent the hard problem must avoid relying on a conceptual framework that invertedly reintroduces the very dualism at its core. By placing the notion of *flesh* at the center of his late work, Merleau-Ponty accomplishes precisely this. Rather than conceiving of constitution as something that is *formed* by a subject to bring about the object of our experience, Merleau-Ponty invites us to recognize both the seer and the seen as co-participants in *a process of mutual enfolding*—a dynamics through and in which seer and the seen *co-emerge through their reciprocal involvement*. Although, as we have noted, we must be careful to not reify flesh by forcing it into conventional categories such as matter, mind, substance, or the like, it remains safe to follow Merleau-Ponty in thinking of flesh as the “means of communication” (1968, 135) between seer and thing—as the “third term” (ibid., 94) from which their very relation arises. Note, however, that qua reversibility thesis, the relation between seer and seen is radically reciprocal. At any point in the process of constitution, an object can transform into a “quasi-subject” (Apostolopoulos 2017, 692), relaying meaning back to the seer in ways that constrain the possible trajectories along which concrete processes of constitution unfold.

To summarize the results of this and the previous section: The strength of Merleau-Ponty’s radical rethinking of constitution, and its significance for the project of radical neurophenomenology, lies in its ability to move beyond the classical view of a subject actively bestowing meaning onto an inert world of things or hyletic data. Instead, constitution emerges as a *dynamic and reciprocal process* in which the seer and the seen, subject and object, co-constitute each other through their mutual involvement. This interplay is *not* reducible to either side, *nor* does it entail that there are first two relata that at a later stage are put in relation to each other, but it is mediated by what Merleau-Ponty calls *“*flesh*”*—a fundamental medium that enables their reversibility and interrelation. Rather than a unilateral act of sense-bestowal, constitution unfolds as a process of *sedimentation and transformation*, in which past and present, activity and passivity, continuously shape and reshape the possibilities of meaning.

In the next two sections, we turn to examples of mild neurophenomenology, and to the two exemplary domains of neurophenomenological inquiry—neuroimaging and phenomenological analysis of research participants’ lived experiences—in order to illustrate how a Merleau-Pontyan understanding of constitution as mutual, embodied co-emergence can inform and reshape concrete research practices in these domains. We will first examine modern brain imaging techniques from a phenomenological perspective, while a discussion of phenomenological analysis of research participants lived experience of a particular issue or condition will follow in a later section. In both sections, we argue that the reconfigured understanding of constitution offers a basis for analyses in and across these domains, and for a dialogue across neurosciences and phenomenological accounts of experience that do not rest with the idea of bridge a perceived gap between “objective” and “subjective” poles. At stake, instead, is a rethinking of the very terms of objectivity and subjectivity, based on the outlined understanding of the flesh, above.

## Brains and coordinate systems

Brain imaging techniques like magnetic resonance imaging (MRI) and functional magnetic resonance imaging (fMRI) have become ubiquitous in diagnostic medicine and biomedical research.^6^ By 2006, approximately 26.6 million MRI examinations were conducted in the US alone (Joyce 2008, p. 5). Given that MRI and fMRI are not only utilized as diagnostic tools but also widely employed in treatment interventions and research, they stand as two of the most prevalent medical imaging techniques.

Despite several interventions from scientists (Huettel 2016, 747), philosophers (Uttal 2011) and STS researchers (Beaulieu 2001; Sturken and Cartwright 2001; Dumit 2004; Joyce 2008), emphasizing that MRI and fMRI are not a traditional photographic techniques such as X-ray and that, especially in the case of fMRI, their evidential support for functional claims may not be as robust as sometimes assumed (Klein 2010), it remains common to characterize these imaging techniques as tools for “opening ‘the black box’ [of] the human mind” (Camerer et al. 2005, p. 53), as providing “another level of vision, exposing the workings of the most hidden” (Kevles 1997, p. 197). Further, when fMRI is presented in some neurophenomenological research, as seen in studies such as those by Modestino on altered states of consciousness in meditation with potential health benefits (2016) or by Cardeña et al. on automatic writing (2023), it is introduced as a method to produce objective visual data, which is subsequently correlated with what is referred to as “phenomenological experience” or “subjective experience” gathered through phenomenological interviews and/or self-reports (Modestino 2016: 131, 129; Cardeña et al. 2023: 4, 3). Following standard scientific publication conventions, methodological discussions regarding fMRI are confined to the Materials and Methods section, where the technical details about signal processing and statistical analysis are provided, and to the Results Section, where the emphasis lies on data interpretation and the subsequent correlation between subjective experience and objective visual fMRI data. Summarizing the fundamental research trajectory, Modestino describes his neurophenomenological approach as combining “first-person experience […] with neuroimaging to *divulge objective brain regions associated with such experiences*” (2016, 128; our emphasis).

Given this portrayal of fMRI, particularly in neurophenomenological studies such as these, there is a risk of succumbing to what has been described as the “myth of transparency” (Joyce 2008) or the “logic of transparent immediacy” (Bolter and Grusin 1999). What this means can be described as follows: Interpreting brain imaging technologies as tools for generating visual data of a pre-given object can reinforce the notion that fMRI images are the outcome of a strictly regimented set of procedures aimed at providing an objective representation of the target system, without traces of subjectivity being involved or acknowledged. The assumption, then, is that these images, if the procedures for producing them are correctly carried out, offer an objective basis with which their subjective-experiential counterparts can be aligned and that this is how subjectivity enters. In formulations that position fMRIs as “external, quantitative measurements”, in contrast to “subjective experiences” (deCharms 2008, 720), there is a risk that “objectivity” gets to be conceived as what remains once all subjective “disturbances” have been removed from the representation of the target system. Modern brain imaging techniques appear to be an especially powerful tool in this regard, not the least because of their assumed ability to substitute the deficiencies of human perception and judgment with statistical, mechanical, and computational tools.^7^

In a recent publication, Carusi and Hoel (2015) contend that this interpretation of fMRI is flawed on several significant grounds. Drawing from a conceptual framework also influenced by Merleau-Ponty’s later work, Carusi and Hoel underscore the significance of abstract coordinate systems in contemporary brain imaging methods. Their core message can be encapsulated as follows:

[fMRI-generated] images are not simply something that you look *at* or *through*—[these] images are something that you *see according to*, something according to which your vision is being formed. This means that [fMRI] images have their own formative capacity, in the sense that they never merely mirror the pregiven but make a real difference to what is discerned and how. Nor do [fMRI] images simply construct their objects by imposing an arbitrary order on them. As Merleau-Ponty insists, imagining involves *complicity* between vision and its objects; through the specific arrangements of vision, technologies and settings, objects *emerge* in different but systematic ways. […] Neuroimaging is at once formative and revealing of neurophenomena. (Carusi and Hoel 2015, 163 − 64)

What Carusi and Hoel are alerting us to is the following: Due to the widespread use of brain imaging in diagnostic medicine and biomedical research, many of the intricate physical, mechanical and mathematical processes underlying fMRI imagine production have been black-boxed through technologization and computerization. To be sure, this is not to deny that the accurate utilization of brain imaging techniques requires considerable technical expertise. However, the very point of standardizing instruments is to hide many technical and scientific intricacies behind user interfaces to enhance the efficiency with which these techniques can be employed. This is not only the case regarding the physics underlying fMRI which, if discussed at all, tends to be greatly simplified. It is also the case regarding the abstract coordinate systems that are necessary to spatially represent fMRI data to make this data practically useful for neuronavigation purposes, for instance in neurosurgery.

Looking at the history of neuronavigation techniques, it is obvious that their evolution has been closely associated with the introduction of new coordinate systems (Enchev 2009). While earlier methods relied on Cartesian and polar coordinates, the Talairach system was the gold standard in fMRI imaging for many years (Lancaster and Fox 2000). Presently, various additional coordinate frames such as MNI space or even subject-specific coordinates are in use, supported by software solutions like FreeSurfer or BrainSuite, allowing for conversion between different systems (Woods 2000; Poldrack et al. 2011).

Why is it important to underscore coordinate systems in neuroimaging, and how does this relate to our earlier discussions concerning Merleau-Ponty? For one thing, as Carusi and Hoel argue, emphasizing coordinate systems in neuroimaging is relevant because it highlights the constructed nature of the visual representations produced by modern brain imagining techniques. As previously discussed, one perspective on instrumentally aided vision is to perceive technology as liberating the perceptual process from subjective influences, thereby yielding an objective representation of the target phenomenon. However, once we realize that individual voxels (the primary dataset representing the blood-oxygen-level dependent contrast measured by fMRI) can only be spatially identified if a suitable coordinate system has been introduced, it becomes evident that transforming raw data into meaningful visualizations is a complex process involving many constructive steps. Seen from this perspective, then, fMRI serves as a fitting instance of Merleau-Ponty’s claim that vision, when properly understood, neither merely records what is already present nor imposes order on the observed (Carusi and Hoel 2015, 152–155). Rather, for vision to occur, seer and seen must enter a “strange system of exchanges” (Merleau-Ponty 1964, p. 125) which fMRI aptly illustrates.

What Carusi and Hoel describe as the “complicity between vision and its object”, then, should not be understood as a simple act of looking at a pre-given image.^8^ Rather, following Merleau-Ponty, “vision” designates a structural relationship through which meaning emerges between embodied perceiver and worldly phenomena. In the case of neuroimaging, the fMRI image does not reveal the brain as it is “in itself”; rather, it stages a mediated encounter in which both the construction of the image and its interpretation are shaped by the interplay between human embodiment, technological mediation, and experimental constraints. From this perspective, brain imaging is not the passive registration of data but an active articulation of the flesh—a process in which the image, the scientist, and the brain all participate in constituting what counts as a neurophenomenon.

Furthermore, recognizing the indispensable role of coordinate frames for the constitution of meaningful spatial data also carries significant implications for our conception of the fundamental idea of scientific objectivity. While the regimented exclusion of subjective “disturbances” is a key motivation driving the development of scientific instruments, this does not imply that the complete exclusion of all subjectivity is either possible or even desirable for achieving objectivity. In fact, the example of fMRI suggests otherwise. Even if, for the sake of argument, we assume that the raw data generated by fMRI is entirely free from any factors related to the perceiving subject, the essential outcome of the preceding discussions is that this raw data remains uninterpretable due to its inability to be spatially identified—a limitation that can only be addressed by implementing a coordinate frame.

Yet, it is at this juncture that we must consider the question what a coordinate system actually is. Although a detailed discussion of this issue is beyond the scope of this paper, several phenomenological studies suggest that the origin of a coordinate system is the most formal representation of the scientist’s lived body, her “zero-point of orientation” (Husserl 1983, 166); the axes of the coordinate system, on the other hand, represent the scientist’s orientation in space.^9^ If this is correct, then this indeed carries significant implications for our conception of objectivity: Whenever we seek to make the data produced by fMRI meaningful by spatially identifying it, a coordinate system must be implemented, and whenever a coordinate system is implemented, a minimal sense of subjectivity reappears in what seemed to be a dehumanized representation of the world. From the perspective of Merleau-Ponty’s later works, recovering this minimal sense of subjectivity is crucial because it shows that fMRI, like any other interaction with our worldly surroundings, is a continuous articulation of the flesh which requires participation of both the seer and the seen.

To summarize this section: Following the later Merleau-Ponty, we propose to understand neuroimaging as a complex process of co-constitution, characterized by reciprocity and mutual enfolding between multiple poles. As Carusi and Hoel aptly point out, fMRI images are neither “something that you look *at* or *through*” (2015, 163), nor are they raw data from which theoretically guided interpretations are constructed. Instead, for neurophenomena to manifest themselves, a complicity is required between competent seers, autonomous machines, and things that allow themselves to be seen in this specific way. Within concrete processes of co-constitution, these poles exist in a reciprocal relation, where any one of them can at any point take on the role of a “quasi-subject”, relaying meaning back to the other poles and constraining the possible trajectories along which the process unfolds. A defining characteristic of this process is that traditional demarcations between objectivity and subjectivity become entirely artificial: traces of subjectivity—in the form of coordinate systems—are embedded at the very core of neuroimaging technology, while the seer’s gaze is *domesticated* through a technologically regimented way of looking. It is only through this “strange system of exchanges” (Merleau-Ponty 1964, p. 125) that flesh articulates itself in the particular form of neurophenomena. Of course, this is not to suggest that fMRI is unreliable or deceptive, nor that neuroscientific data lacks objective significance. Rather, the point is that neuroimaging is embedded in an interpretive framework shaped by embodied, instrumentally mediated engagement—what Merleau-Ponty refers to as the *flesh*. In this sense, objectivity does not disappear but is reframed as a mode of disclosure that is always situated, mediated, and dependent on contingent methodological configurations.

## Research participants’ lived experiences

As our aim is to show how our framework matters for *both* modern brain imaging techniques and phenomenological analysis of research participants’ lived experience of a particular issue or condition, we also turn to analyses that combine phenomenological philosophy with qualitative research such as interviews, hereafter referred to as qualitative phenomenological philosophy. When so doing, we follow the steps of the section above: we briefly introduce qualitative phenomenological philosophy, note what we see as lacking in mild neurophenomenological studies on the “subjective experience” of research participants, and discuss what difference it would make if analyses of research participants’ lived experiences instead where done based on the radical neurophenomenology insights that we argue for in the article.

While brain imaging techniques have become integral parts of both diagnostic medicine and biomedical research in the 21st century, qualitative research of lived experiences has a much longer history. All such research does not engage with phenomenological philosophy. On the contrary, this is only the case for a small fraction of this kind of research. However, to the extent that neurophenomenology claims to engage with subjective experience of research participants from within phenomenological philosophy, this kind of research becomes particularly relevant. Further, qualitative phenomenological philosophy is a growing area also outside neurophenomenological discussions, where scholars in phenomenological philosophy and/or critical phenomenology combine performing qualitative research with phenomenological tools; they conduct and analyse interviews, with a phenomenologically philosophical aim (see, for example, Høffding and Martiny 2016; Køster and Fernandez 2021; Stanier 2022; De Boer and Zeiler 2024; Vēgners et al. 2025). Some such scholars engage in combining qualitative research and phenomenological philosophy with a focus on more or less invariant structures of experience (Petitmengin and Bitbol 2009), while others argue for the study of first-person lived experiences in “phenomenologically grounded qualitative research” with a focus on altered modes of being-in-the-world (Køster and Fernandez 2021). Still others analyse research participants’ lived experiences with an eye for altered modes of being-in-the-world and contingent yet constitutive structures of experiences such as socio-cultural understandings and norms about particular bodies or illnesses, asking how these help shape people’s very ways of perceiving and experiencing themselves, their bodies and their environment (De Boer et al. 2019; Slatman 2014; De Boer and Zeiler 2024).

The qualitative phenomenological philosophy that we argue for, in this article, aligns with the later Merleau-Ponty’s concept of constitution. This makes it different from qualitative research that make use of phenomenological concepts or labels itself phenomenological but doesn’t attend to constitution in the sense of the later Merleau-Ponty. It also makes it different from common work in mild neurophenomenology. To keep to the examples in the section above (Cardeña et al. 2023; Modestino 2016), such neurophenomenological studies describe themselves as concerned not only with neuroimaging but also with the “subjective” and “experiential” side, and state that they aim to identify patterns, themes and features across participants’ interviews or self-reports. However, they do so with little or no attention or acknowledgment being given to the constitutive interconnectedness of subject and object *as part of* the analysis. Even though the term “subjective” or “subjectivity” is used, it is used in a way that does not acknowledge or ask how the sense or meaning of something is constituted in experience.

We see this as concerning: when constitution in the sense outlined via the later Merleau-Ponty is not acknowledged, neurophenomenological studies risk reproducing an understanding of subjectivity as neatly set apart from anything “objective” and/or as possible to set apart from its environment. Our argument is therefore the same as in relation to the “objective” side of neurophenomenology, i.e., the use of brain imaging techniques, but this time focused on the understanding and analysis of narrated or written down experiences by participants in neurophenomenological studies. As argued throughout this article, *not* to attend to constitution is to miss a phenomenological philosophy insight and this matters for the understanding of the knowledge that is being produced, just as it did in the section on MRIs and fMRIs. If one does not acknowledge the constitution, and in parallel with the reasoning on how the procedures for producing (f)MRIs gloss over the role of subjectivity in the minimal sense of a coordination system as a representation of the scientist’s “zero-point of orientation” and not recognize the relation between subject and object, and subjectivity and objectivity, then the “subjective” side within mild neurophenomenology may be understood in an equally reduced way: as if it was possible to aptly describe and understand an experience, as an object of study, without acknowledging the role of the subject-object relation for this very experience. This can be put even stronger through the discussion via the later Merleau-Ponty: to acknowledge the role of reversibility and to overcome the dualistic frameworks that have constrained our thinking, we need to abandon the understanding of the subjective as what is *not* objective, and the body as what is *not* the world, and ask about the co-constitution of body and world, the subjective and the objective.

This means that it is not enough only to refer to “subjective experiences” in neurophenomenological studies, at least if the phenomenology part of the neurophenomenology is intended to refer to phenomenological philosophy of the kind outlined in this article. What is needed, instead, is a qualitative phenomenological analysis of research participants’ lived experiences of a particular issue, condition or situation that asks how the sense or meaning of it is constituted in embodied experience through a relation of reversibility between subject and object. This is to study subjectivity from within a non-dualistic framework that moves away from attributing activity to the “subject” side and passivity to the “object” side; it is to acknowledge the role of the constitutive interconnectedness of subject and object for both, and for rethinking the idea of “sides”.

What, then, would this mean, concretely, for phenomenological studies of research participants’ first-person lived experiences? First, just as brain imaging is not just used in neurophenomenology, qualitative phenomenological philosophy analysis of research participants’ lived experiences are also not just performed in a neurophenomenological framework. In both cases, insights can be gained from broader phenomenological discussions of the particular method. However, to our knowledge, no mild neurophenomenological study is explicitly based on the later Merleau-Ponty’s concept of constitution. Further, and unfortunately, even though the bulk of qualitative phenomenological philosophy analyses of patients’ lived experience of, for example, illness and pain, is growing, such research has so far (to the best of our knowledge) not been *explicitly* based on this (later Merleau-Pontian) understanding of constitution (even though some such research, arguably, is implicitly informed by it or can be aligned with it).

For this reason, we ask the reader to envision the following: A qualitative phenomenological philosophy inquiry is performed with the aims of investigating and offering accounts of interviewees’ lived experiences of a particular illness, with a focus on overall alterations in their modes of being-in-the-world, (contingent yet constitutive) structures of experience, and on how their sense of self, embodiment, others, time, and the world manifest in their experience. The inquiry involves interviews, in which interviewees are invited to narrate how they fell ill, what this was like, what happened, what it meant and felt for them – including how they experienced themselves, their bodies, and their ways of engaging with others and their environment, within this experience. The interviews are also designed to allow for narrated experiences of sensations, senses, and is not restricted to patients’ experiences of what might be termed as symptoms. The qualitative phenomenological analysis can then be performed with a focus on how the illness alter interviewees’ experience of their bodies and the world – how bodies and world manifest in the illness experience – and offer an account of how the illness experience is constitutive and constituted by particular socio-culturally shared and prevailing biomedical frameworks, and/or understandings (or uncertainties, taboos, contestedness) about the particular illness.

So far, this description is broadly in line with how a qualitative phenomenological study of lived experiences of testing for dementia proceeded (Zeiler et al. 2024), and we will briefly engage with this study to discuss, concretely, what *kinds of questions would be central* for a qualitative phenomenological analysis *if it were based* on the understanding of constitution that we argue for, be it in neurophenomenology or within phenomenology of medicine more generally. Please note that this study did not explicitly engage with the later work of Merleau-Ponty but is compatible with such an approach. The study was a small-scale study of lived experiences of patients who had undergone testing for dementia, and the analysis resulted in an account of a felt sense of entering an affectively charged “grey zone of aging between ‘healthy’ aging and aging with a disease” (Zeiler et al. 2024: 673). To enter this grey zone, entailed experiencing oneself as neither fully healthy nor as having a disease, *and* as needing to negotiate and live this ambiguity as a disease diagnosis was not (yet) given but the disease nonetheless loomed as a real possibility for the self. Entering the grey zone, the analysis showed, entailed certain ways of being part of and connected to the world, “in *worrying* ways” (Zeiler et al. 2024: 672). The affectively charged ambiguity helped shape – constitute – people’s very ways of perceiving themselves, when in the grey zone, and they could become “stuck” in a reflective mode of attending to one’s embodiment and to signs of cognitive deterioration (Zeiler et al. 2024: 673).

Now, to attend to how the sense or meaning of being tested for dementia is constituted in experience, in this case, implies examining how socio-cultural constitutive yet contingent understandings about dementia are integral to and help shape interviewees’ very ways of perceiving and making sense of these tests. This was done in this study, and it implied investigating how affective qualities shaped and were parts of the self’s mode of being-in-the-world, when having entered the “grey zone.” However, to engage in qualitative phenomenological philosophy of lived experience of illness or pain (or, above, experiences of neither being fully healthy nor having a disease) from within the later Merleau-Ponty’s conception of constitution, would require a further step of explicitly asking how *the sense or meaning of the illness is constituted in embodied experience through the very relation of reversibility and constitutive interconnectedness of self and world.* This would entail a distinct framework when performing interviews and doing the qualitative phenomenological philosophy analysis. It would entail attention not only, for example, to narrated experiences of alterations in the interviewees’ overarching way of being and experiencing others and the world, but an explicit attention to and discussion of how the self-world interconnectedness constitutes embodied experience. And while this is implicitly already part of some studies within qualitative phenomenology, it would need to be rendered explicit in the kind of study that we propose.

Moreover, the attention to constitution in the sense of the later Merleau-Ponty carries implications not only for our conception of objectivity (as discussed in relation to MRIs), but also for the conception of subjectivity. In parallel to the earlier reasoning, one may say that a minimal sense of “objectivity” always already is present and integral to what gets to be described as the “subjective” side. Note that this entails a move away from both the understanding of objectivity as that which is left when all subjectivity is removed and that of subjectivity as not entailing any objectivity. The *senses* of these terms are, now, different.

In the qualitative phenomenological analysis, how the world manifest in experience and is narrated in the interview cannot be understood independently from “the subject through which and for which it is object” and “the subject reveals itself only through the objects in which it is engaged,” to make use of Beauvoir’s apt description of one of the central phenomenological insights (160). Or, to keep with Merleau-Ponty’s earlier example: not only trees, but any situation, any lived experiences of the world and things in it, are always already affected by the peculiarities of our situated gaze, the experiential history that is built into our perceptual horizon, and by the constitutive interconnectedness of subject and object, self and world. This becomes central for doing qualitative phenomenological philosophy in ways that are informed by insights from the later Merleau-Ponty’s understanding of constitution, and for inquiry into research participants’ lived experiences in studies that self-identify as in line with radical neurophenomenology.

## How this matters for medicine and health care

The aim of this paper was to build on existing work in radical neurophenomenology, but to improve it conceptually and in terms of the concrete impact such a reconceptualization would have. On a conceptual level, our interest was to develop a concept of constitution that can accommodate neurophenomenological research methodologies without presupposing the very dualism radical neurophenomenology seeks to prevent. We have argued that Merleau-Ponty’s late philosophy offers the resources to explicate a concept of constitution that avoids being caught up in the dualistic logic and discussed the concept of flesh and the generalized reversibility thesis that undermines the dogmatic attribution of activity to the “subject”-side and passivity to the “object” side in all our worldly involvements. We have also discussed how our framework could influence concrete work in neurophenomenology, by exploring two examples representing typical research methods utilized and “correlated” in mild neurophenomenological studies. Taking the reasoning on constitution via the later Merleau-Ponty seriously, entails acknowledging that this requires a rethinking of both “objectivity” and “subjectivity.”

Let us consider one more question: A critic might argue that even if our arguments are accepted, it remains unclear how our reconceptualization would impact the day-to-day practice of neuroimaging or qualitative phenomenology. Would a phenomenologically attuned neuroscientist apply any of her methods differently? And does a phenomenologist engaging in qualitative phenomenology that acknowledges and builds on insights from the later Merleau-Ponty do this differently from other researchers engaging with qualitative research?

Our answer to this question is twofold: On the one hand, with a view on neuroimaging, we do not expect neuroscientists to apply their methods differently if by “apply their methods” we mean the practical aspects of operating scanners or controlling unwanted noise. However, what would change is the *mindset* with which researchers approach their own research results, especially when these results are then brought in contact with findings that rely on an entirely different set of methodological tools. Recognizing that even our most advanced tools are, in essence, constitutive practices that strive to continuously articulate the flesh heightens our awareness of the presuppositions that are invariably present in every cognitive interaction with our environment. This includes not only complex experimental setups but also seemingly simple acts of perception—as discussed earlier in the example of the trees—thereby underscoring that the ontological conditions of scientific objectivity are continuous with those structuring everyday embodied experience. This understanding should not be misconstrued to suggest that there are no qualitative differences among various cognitive practices, or that certain practices are not more adequate in dealing with specific types of phenomena than others. However, once we acknowledge the constitutive nature of these practices, their evaluation can only be conducted through a continuous iterative process between these practices, rather than in comparison to a supposed “external” standard like a classical concept of objectivity. Compared to the classical concept of objectivity, this entails a major shift.

Hence, when asked about the pragmatic gain of our discussion, we align with Bitbol in aiming to effect change on an existential rather than merely a purely intellectual or practical level (Bitbol 2012). This means that researchers who embrace the suggested framework will adopt a perspective that extends beyond individual decisions regarding research design or data interpretation—the transformation brought about by a Merleau-Pontyan stance impacts the very core of our most primordial situation in the world. Further, for the “subjective” side of neurophenomenology, taking constitution seriously would have implications for the very analysis of lived experience. It would require the researcher to acknowledge and be attentive to constitution – in the sense outlined in this article – and the constitutive interconnectedness of self and world, and ask questions about how meaning is constituted in embodied experience through the relation of reversibility and constitutive interconnectedness of selves and world, *as part*s of the analysis. This should be done in neurophenomenological as well as phenomenology of medicine inquiries that builds on insights from the later Merleau-Ponty.

Finally, what are the implications of this for health research or research of relevance for medicine and health care that has sought to create a dialogue across the neurosciences and phenomenology, without naturalizing the latter? We contend that the neurosciences and phenomenology (including qualitative phenomenological philosophy) have much to gain from being brought into such dialogue for the sake of a nuanced understanding of conditions, in ways that make room for insights from different perspectives. However, to do so from within the work of the later Merleau-Ponty requires acknowledging the co-constitution of the objective and the subjective. It requires a move away from the idea of creating a bridge between “objective” and “subjective” domains, where neuroscientific knowledge is assumed to contribute objective knowledge about a particular issue or condition, and phenomenological inquiry understood as offering a way to identify basic features of the experience of it, along the lines of quests for correlations.

Instead, reasoning from within radical neurophenomenology opens for other kinds of inquiries. It provides shared ground for *both* brain imaging analysis and qualitative phenomenological inquiry of particular issues or conditions, in non-reductive ways. It opens for a dialogue across knowledge and insights from within biomedical science and qualitative phenomenology of research participants’ – such as patients’ – lived experience of a particular condition, in ways that shifts away from the focus on bridging a gap between “objective” and “subjective” poles.

This also takes us back to where we started: radical neurophenomenology, if applied and made use of in the context of biomedicine and phenomenology of medicine – and in interdisciplinary health research across these – problematizes the idea of objectivity and subjectivity as neatly set apart, and the related idea of third-person and first-person perspectives as equally neatly set apart. To take radical neurophenomenology seriously is not simply to repeat concerns about the dominance of a third-person perspective of the natural sciences as the primary basis for disease conceptions and diagnostic methods, at the expense of phenomenological inquiry into a lived experiences of illness and pain. It requires a rethinking and re-conceptualizing of the relation between subjectivity and objectivity, that thoroughly moves away from the Cartesian reasoning that has shaped modern medicine (see Leder 1992). In addition, if one engages in interdisciplinary research across biomedicine and phenomenology, then this entails a call for such research that problematize the established conceptualizations of third-person “objective” vs. first-person “subjective” framings in knowledge production that aims for a nuanced understanding of a particular issues or condition, and that acknowledge the intrinsically intertwined, reversible subject-object relation that shape and reshape the very possibilities of meaning.

## Footnotes

1 Further, seeing a difficulty in obtaining “reliable” experiential data, Varela emphasized the importance of trained subjects as collaborators in generating fine-grained phenomenological reports. For discussions of Varela’s original vision for neurophenomenology, cf. Depraz 2002 and the papers collected in the special issue edited by Depraz.

2 We use the words “constitution” and “co-constitution” synonymously. Typically, when philosophers consciously use “co-constitution”, it is to disentangle the concept from the idea of a worldless subject whose sense-bestowing activity is primary for the process of world-manifestation. However, since the point of Sect. 2 is to distance ourselves from such an interpretation, “constitution” seems admissible.

3 Cf., for more details on Husserl’s concept of horizonal intentionality, Smith 2007a; Chap. 6.

4 Cf., for a discussion of common misrepresentations of Husserl within parts of the Merleau-Ponty scholarship, Zahavi 2002.

5 It would go beyond the scope of this paper to comment in detail on whether this charge of intellectualism is justified. For critical discussions of this point, cf., e.g., Zahavi 2002; Smith 2007.

6 As one reviewer has pointed out, their role is quite different in research and diagnostics. We agree. However, as will become clear, whether an fMRI is used to investigate working memory in a research setting or to localize a lesion in a clinical context, the reliance on coordinate systems as constitutive structures of objectivity remains the same.

7 To be clear, our critique here is not directed at philosophers like Thompson, Bitbol, or Petitmengin, whose work on radical neurophenomenology explicitly emphasizes the entanglement of subjectivity and objectivity. Rather, our concern is with empirical studies that adopt the label “neurophenomenology” while continuing to frame methods such as fMRI as a method for producing objective data to be straightforwardly correlated with subjective reports, without critically engaging with the ontological and methodological assumptions that such a framing implies.

8 While fMRI data is often presented visually, it is better understood as a spatialized statistical mapping of activity changes, projected onto a brain-like anatomical structure. In this sense, fMRI is closer to a graph than to a literal picture. However, Merleau-Ponty’s broader concept of *vision* accommodates such mediated renderings, provided they are understood as part of a constitutive structure in which the seer and the seen are co-implicated.

9 The view that coordinate systems play a constitutive role in the formation of physical objectivity as the most formal representation of the physicist’s embodied location is inspired by the mathematician and theoretical physicist Hermann Weyl who was strongly influenced by Edmund Husserl’s phenomenology. See, for a discussion of Weyl’s ideas and their relation to phenomenology: Ryckman 2005.
